# Unbalanced biparental care during colony foundation in two subterranean termites

**DOI:** 10.1002/ece3.4710

**Published:** 2018-12-30

**Authors:** Lou Brossette, Joël Meunier, Simon Dupont, Anne‐Geneviève Bagnères, Christophe Lucas

**Affiliations:** ^1^ Institut de Recherche sur la Biologie de l'Insecte (UMR7261) CNRS – University of Tours Tours France; ^2^ CEFE, CNRS UMR5175, Univ. Montpellier, Univ. Paul Valéry Montpellier 3, EPHE, IRD Montpellier France

**Keywords:** division of labor, foundation, parental care, social behavior, task allocation, termites

## Abstract

Parental care is a major component of reproduction in social organisms, particularly during the foundation steps. Because investment into parental care is often costly, each parent is predicted to maximize its fitness by providing less care than its partner. However, this sexual conflict is expected to be low in species with lifelong monogamy, because the fitness of each parent is typically tied to the other's input. Somewhat surprisingly, the outcomes of this tug‐of‐war between maternal and paternal investments have received important attention in vertebrate species, but remain less known in invertebrates. In this study, we investigated how queens and kings share their investment into parental care and other social interactions during colony foundation in two termites with lifelong monogamy: the invasive species *Reticulitermes flavipes* and the native species *R. grassei*. Behaviors of royal pairs were recorded during six months using a non‐invasive approach. Our results showed that queens and kings exhibit unbalanced investment in terms of grooming, antennation, trophallaxis, and vibration behavior. Moreover, both parents show behavioral differences toward their partner or their descendants. Our results also revealed differences among species, with *R. flavipes* exhibiting shorter periods of grooming and antennation toward eggs or partners. They also did more stomodeal trophallaxis and less vibration behavior. Overall, this study emphasizes that despite lifelong monogamy, the two parents are not equally involved in the measured forms of parental care and suggests that kings might be specialized in other tasks. It also indicates that males could play a central, yet poorly studied role in the evolution and maintenance of the eusocial organization.

## 1 INTRODUCTION

Parental care is a taxonomically widespread phenomenon across animals (Klug & Bonsall, 2010; Korb, Buschmann, Schafberg, Liebig, & Bagnères, 2012; Wong, Meunier, & Kölliker, 2013). It can last from a few days to several years, be performed before and/or after the emergence of juveniles and involve either the mother, the father or both parents (Smiseth, Kölliker, & Royle, 2012). From mammals to insects, parental care can take multiple forms, such as egg and offspring attendance, nest building and burrowing and food provisioning (Smiseth et al., 2012). All these forms typically provide benefits to offspring by enhancing offspring survival, growth, and/or quality, as well as by improving their lifetime reproductive success (Klug & Bonsall, 2014; but see Kramer et al., 2017). However, investing into parental care may also go along with costs for parents. That is because it can entail an exaggerated loss of energy, as well as an increased risk of pathogen exposure and predation during offspring attendance, which all may ultimately curtail their survival rate and capability to invest into future reproduction (Alonso‐Alvarez & Velando, 2012). The evolution of parental care therefore requires that its costs remain lower than its associated benefits for each family member.

For parents, one way to reduce the costs of parental care is to share them with the other parent. The presence of two parents with offspring has been reported in numerous birds, cichlid fishes, primates, and a few insects (Balshine, 2012; Trumbo, 2012). Although this mutual presence is typically associated with biparental care, a sexual conflict between mothers and fathers over their respective investment into care often emerges during family life, as each parent can maximize its own fitness by selfishly minimizing its investment into cares (Lessells, 2012). Such a selfish strategy allows males, for instance, to increase their investment into the search of additional partners and thus to maximize the number of offspring produced during a single reproductive season, while it allows females to reallocate their saved energy into future reproduction (Smiseth et al., 2012). The tug‐of‐war between mothers and fathers over parental investment has been shown to generally lead to a disequilibrium, during which one parent exhibits a lower investment compared to the other, while this latter does not fully compensate for this reduction (Harrison, Barta, Cuthill, & Szekely, 2009).

Although most studies on sexual conflict explore its resolution in species where parents can do extra pair copulations and/or have novel mating partners at each reproductive season (Jennions, Kahn, Kelly, & Kokko, 2012), the expression and organization of biparental care remain unclear when the lifetime fitness of each parent tightly relies on its partner's. This is the case, for instance, in the biparental family units often present in termites (Kramer & Meunier, 2018; Wilson, 1971). In this eusocial insect, mothers (queens) and fathers (kings) form pairs quickly after they reach adulthood and remain together during their entire lives, which can last decades (Boomsma, 2009). Each couple typically lives in a dark nuptial chamber at the center of the colony, where queens produce eggs and kings regularly inseminates queen(s); kings and queen having no direct contact with foreign individuals (Hartke & Baer, 2011). Because the termite royal couple has no opportunity of extrapair copulation, it has long been thought that parental care is equally shared between queens and kings especially at colony foundation (Bignell, Roisin, & Lo, 2011; Nalepa & Jones, 1991; Shellman‐reeve, 1997). However, empirical support of this claim remains scarce (Rosengaus & Traniello, 1991; Shellman‐Reeve, 1990).

In this study, we investigated how termite queens and kings share their investment into social interactions, as well as whether this share depends on the developmental stage of their offspring. Using an experimental setup allowing non‐invasive and fine‐scaled behavioral observations (Brossette et al., 2017), we analyzed the expression of grooming, antennation, trophallaxis (proctodeal and stomodeal), and body‐shaking by queens and kings over the six first months of their colony foundation. Because we aimed at taking a broader perspective and exploring whether this biparental organization was species specific, we used two species of subterranean termites: the invasive *Reticulitermes flavipes* (Kollar, 1837) and the native *R. grassei* (Clément, 1978). If sexual conflict between males and females over their respective investment in parental care is absent in these species, we expected queens and kings to express a similar level of grooming, antennation, and trophallaxis toward their offspring. Note that body‐shaking is a behavior that has been frequently reported in termites, but for which the role is still unclear (Funaro, Böröczky, Vargo, & Schal, 2018; Whitman & Forschler, 2007). Our study will thus also provide novel insights into our understanding of its expression and function during colony foundation.

## 2 MATERIAL AND METHODS

### 2.1 Sampling and crossings

We investigated the behaviors of newly produced queens and kings originating from a total of four colonies of *R. flavipes* and four colonies of *R. grassei. *The workers, nymphs (i.e., future queens and kings), and soldiers of each of these colonies were field sampled in March 2014 in pine forests on Oléron Island in France and immediately transferred into plastic boxes (18 × 24 × 9.5 cm) with their own nest material and moistened sand (Brossette et al., 2017). These colonies were 100 m away from each other for *R. grassei* and 300 m for *R. flavipes*, that is, distances that typically ensure colony independence (Perdereau, Bagnères, Dupont, & Dedeine, 2010). Back in the laboratory, these field‐sampled colonies were maintained under standard conditions (80% relative humidity, 26°C, 13.5 L/10.5 D cycle) until nymphs became reproductive adults. To prevent uncontrolled sib‐mating, each colony was checked twice a day to collect the newly produced winged alates (females and males, i.e., future new queens and kings) and to transfer these individuals into sex‐specific new plastic boxes (50 mm diameter; Starpack) containing moistened pure cellulose paper (47 mm diameter; Whatman, GE Healthcare; Brossette et al., 2017). Seven days after the emergence of the first winged alate, virgin males were paired with unrelated virgin females. To limit the risks of mating incompatibility between colonies, we paired individuals following 12 intercolonial combinations (later called cross ID), which were each replicated from 3 to 9 times (later called pair ID). This led to a total of 70 and 86 experimental pairs of *R. flavipes* and *R. grassei*, respectively. Each pair was then transferred to an experimental glass case allowing detailed behavioral observations (Brossette et al., 2017) and containing a food source composed of a pure cellulose disk (90 mm in diameter; Whatman, GE Healthcare) supplemented with a solution composed of mineral salts, vitamins, and nitrogen (Argoud, Mocotte, & Sternalski, 1982). Over the subsequent six months of experiment, all pairs were maintained under standard laboratory conditions (80% relative humidity, 26°C) and complete darkness. Humidity was controlled with the use of potassium nitrate wells (35 ml KNO_3_/100 ml H_2_O; Thermo Fisher Scientific).

### 2.2 Behavioral recording

Over the six months of the experiment (from May to October), 12 pairs per species were randomly selected every 2 weeks to be video‐recorded (Sony HDR CX700V). The chambers where the royal couple were settled with eggs and larvae were video‐recorded for 30 min (after a five‐min resting time, as the experimental glass cases were moved to the recording setup), under controlled environment (80% RH, 26°C) and total darkness using infrared lights (940 nm wavelength, 15 LEDs of 26 mm diameter, Kingbright). The presence of eggs and larvae in the royal chamber were assessed. Note that because the parents were the focal individuals, we discarded three videos with missing reproductives from the statistical analyses. The resulting videos were analyzed with the freeware Boris v3.0 (Friard & Gamba, 2016) to quantify parental care behaviors between parents and between parents and descendants. This allowed us to disentangle behaviors that are specifically directed toward offspring (i.e., parental care) from behaviors that are directed toward all family members. In these analyses, donor individuals were defined as individuals expressing the behavior (queen or king), while recipients were defined as individuals receiving the behaviors (defined as either partners, eggs or descendants—this latter including larvae, nymphs, and workers). The recorded behaviors were (a) grooming and antennation (i.e., any contact from the head of a donor toward a recipient), (b) trophallaxis (either proctodeal or stomodeal, i.e., anal‐to‐mouth or mouth‐to‐mouth fluid transfer, respectively), and (c) body‐shaking (rapid back and forth movement of the whole body with no contact with the substrate). Note that this latter behavior is not directed toward any recipient (Whitman & Forschler, 2007). Other behaviors were observed, but discarded from this study because they were not directly involved in parental care (e.g., dejections, selfgrooming, copulations, and food intake). For each video, queens and kings were discriminated by measuring the size of their seventh sternite (Zimet & Stuart, 1982). Videos were processed following a double blind process during recording and reading (Gamboa, Reeve, & Holmes, 1991).

### 2.3 Statistical analyses

The total duration of antennation and grooming behaviors (together) was analyzed using a general linear mixed effects model (LMM), in which the explanatory factors were the donors (Queens/Kings), the recipients (Partner/Offspring), the species (*R. flavipes*/*R. grassei*), and the developmental stage of the offspring (Eggs/Descendants). To interpret the resulting significant triple interaction involving recipients, species, and offspring developmental stage (see Section 3), the dataset was then split per developmental stage and the two resulting subsets were used to conduct two additional LMMs with the same explanatory factors (without the developmental stage factor). The observation of at least one type of trophallaxis (presence/absence) and the total duration of trophallaxis (when observed) were then tested using a generalized linear mixed effect model (GLMM) with binomial error distribution and a LMM, respectively. In these models, the explanatory factors were the donor, the recipient, the species, and the type of trophallaxis (proctodeal/stomodeal). Note that these models were restricted to the dataset where descendants were present, because trophallaxis is not possible toward eggs. Finally, the observation of at least one body‐shaking (presence/absence) and the total number of body‐shaking (when observed) were tested using a GLMM with binomial error distribution and a LMM, respectively. In these models, the explanatory factors were the donors, the species, the presence of eggs, and the presence of descendants.

In all the above statistical models, the cross ID and the pair ID (nested into the cross ID) were included as random factors to control for the fact that several kings and queens came from the same field colonies. The date of each video were also included as a random factor to control for the fact that parental behaviors may change over time, while providing an overview of the different behaviors over the six‐month recording (i.e., the main goal of this study). To fit with homoscedasticity and normal distribution of model residuals, the total duration of antennation and grooming behaviors were log(+1)‐transformed, while the total duration of trophallaxis and the total number of body‐shakings were log‐transformed. All GLMMs with binomial error distribution were fitted using the “cloglog” link‐function to correct for the unbalanced representation of 1 and 0 (Crawley, 2012). All models were first tested with all possible interactions among explanatory variables and were then simplified step‐by‐step by removing the non‐significant interactions (all *p* > 0.08). Note that some non‐significant interactions are reported in the results to allow direct comparison between analyses, but their removal induces no qualitative changes. When required, we conducted post hoc pairwise comparisons within each model using model contrasts based on estimated marginal means. When appropriate, non‐significant factors were pooled in the presented figures. All analyses were performed using the software R v3.4.3 (www.r-project.org) loaded with the packages *lme4*, *car,* and *emmeans*.

## 3 RESULTS

**Figure 1:**
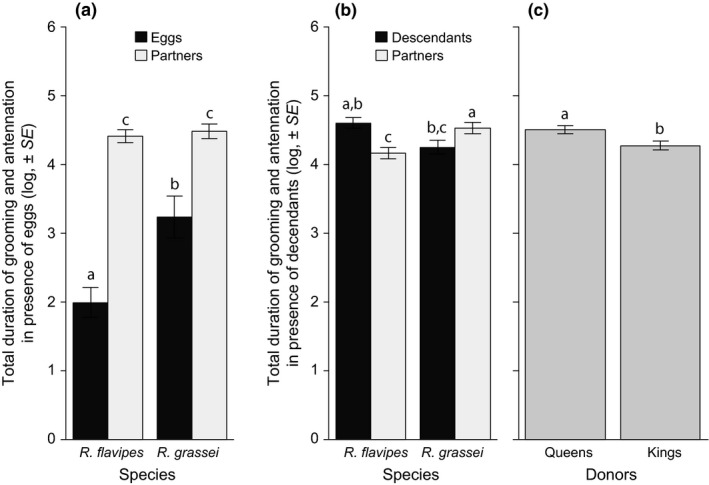
Effects of species (*R. flavipes*/*R. grassei*), recipients (Partner/Offspring), and donors (Queens/Kings) on the total duration of allogrooming and antennation either in the presence of eggs (a) or in the presence of descendants (b, c). When the factors showed no significant interactions (see tables), they were pooled to better represent the statistical results. Bars represent mean values of the log(+1)‐transformed total duration ±*SEM.* Different letters refer to *p* < 0.05

Grooming and antennation were overall present in 86.3% of the movies. The total duration of grooming and antennation depended on a triple interaction between species (*R. flavipes* or *R. grassei*), recipients (offspring or partner), and offspring developmental stage (eggs or descendants; LR $\chi_{1}^{2}$ = 26.0, *p* < 0.0001). In the presence of eggs, *R. grassei* and *R. flavipes* adults spent the same amount of time grooming and antennating their partners (Table 1; Contrast, *p* = 0.9203), whereas *R. grassei* adults spent more time grooming and antennating their eggs compared to *R. flavipes* adults (Contrast, *p* = 0.0247; Figure 1a). Nevertheless, both *R. grassei* and *R. flavipes* adults spent overall more time grooming and antennating their partner than their eggs (Table 1, Figure 1a). Conversely, in the presence of descendants, *R. flavipes* adults spent more time grooming and antennating descendants than partners (Table 1; Contrast, *p* = 0.0002), whereas *R. grassei* adults spent more time grooming and antennating partners than descendants (Contrast, *p* = 0.0091; Figure 1b). During that period, the total time spent grooming and antennating was higher in queens compared to kings, but independent of the species and the type of recipient (Table 1, Figure 1c).

**Table 1:** Effects of recipient, donor and species on total duration of allogrooming/antennation when (a) eggs or (b) descendants were present

	(a) When eggs are present		(b) When descendants are present	
	LR $\chi_{1}^{2}$	*p*	LR $\chi_{1}^{2}$	*p*
Recipient	95.4	**0.0000**	0.4	0.5524
Donor	0.2	0.6516	8.7	**0.0032**
Species	1.6	0.2003	0.1	0.7681
Recipient:Species	9.7	**0.0019**	20.5	**0.0000**

Note

Significant *p*‐values are in bold.

**Figure 2:**
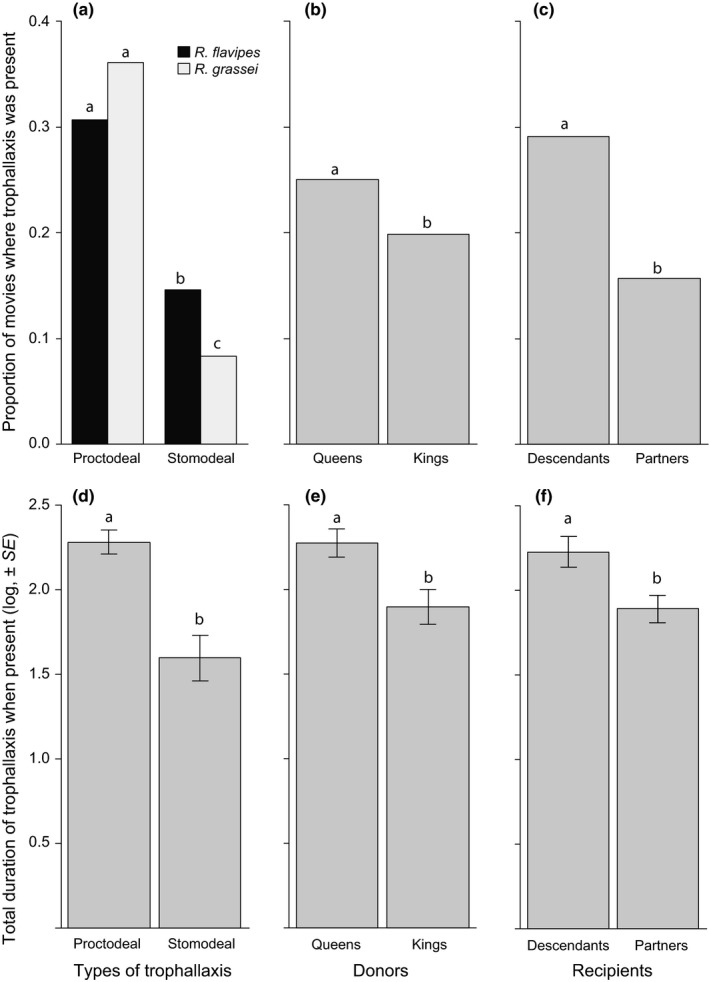
Effects of species (*R. flavipes*/*R. grassei*), recipients (Partner/Offspring), donors (Queens/Kings), and types (Proctodeal/Stomodeal) on the observation (a–c) or total duration of trophallaxis (d–f). When the factors showed no significant interactions (see tables), they were then pooled to better represent the statistical results. Bars represent proportion of movies (a–c) or mean values of the log‐transformed total duration ±*SEM* (d–f); Different letters refer to *p* < 0.05. Note that these models were restricted to the dataset where descendants were present, because trophallaxis is not possible toward eggs

At least one of the two types of trophallaxis (proctodeal and stomodeal) was present in 22.4% of the movies. Stomodeal trophallaxis was more likely to be observed in *R. flavipes* compared to *R. grassei* (Table 2; Contrast, *p* = 0.0430), whereas this difference was absent for proctodeal trophallaxis (Contrast, *p* = 0.2655; Figure 2a). Independent of the species and its type, trophallaxis was more likely to be expressed by queens than kings (Table 2; Figure 2b,e) and more likely to be received by descendants than partners (Table 2; Figure 2c,f). When at least one type of trophallaxis was observed, queens spent more time performing trophallaxis than kings (Table 2), descendants received trophallaxis for a longer total time compared to partners (Table 2) and proctodeal trophallaxis was overall expressed longer than stomodeal trophallaxis (Table 2). The total duration of trophallaxis was independent of any interaction among donors, recipient, and type of trophallaxis (all *p* > 0.0975).

**Table 2:** Effects of recipient, donor, species and types of trophallaxis on (a) the presence of at least one trophallaxis event and on (b) the total duration of trophallaxis when present

	(a) Presence/absence		(b) Total duration when expressed	
	LR $\chi_{1}^{2}$	*p*	LR $\chi_{1}^{2}$	*p*
Recipient	24.8	**0.0000**	10.4	**0.0013**
Donor	4.6	**0.0321**	7.9	**0.0051**
Species	0.0	0.9445	0.8	0.3762
Types of trophallaxis	56.2	**0.0000**	24.6	**0.0000**
Species:Types of trophallaxis	6.8	**0.0091**	1.9	0.1682

Note

Significant *p*‐values are in bold.

**Figure 3:**
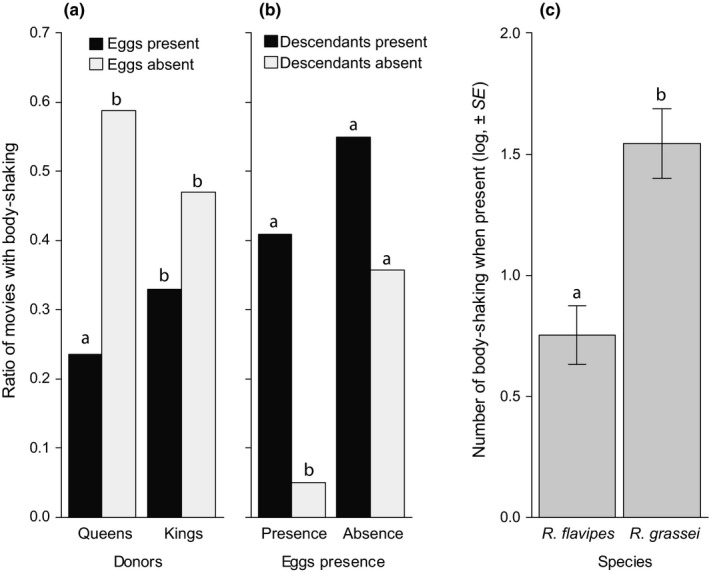
Effects of donors (Queens/Kings), eggs presence, descendants presence, and species (*R. flavipes*/*R. grassei*) on the observation (a,b) and total number (c) of body‐shaking. When the factors showed no significant interactions (see tables), they were then pooled to better represent the statistical results. Bars represent the ratio of movies with body‐shaking (a,b) or the log‐transformed total number of body‐shaking when present ±*SEM* (c); Different letters refer to *p* < 0.05. Note that no recipients were assigned for this behavior

Finally, body‐shaking was observed in 39.2% of the movies. The observation of at least one body‐shaking event depended on double interactions both between donors and eggs presence (Table 3) and between eggs and descendants presence (Table 3). In particular, queens were less likely to perform body‐shaking in the presence compared to in the absence of eggs (Figure 3a; Contrast, *p* = 0.0339), whereas this effect was absent in kings (Figure 3a; Contrast: *p* = 0.9146). Conversely, queens and kings were overall more likely to perform body‐shaking in the presence compared to absence of descendants, but only in the presence of eggs (Figure 3b; Contrasts: eggs presence, *p* = 0.0023; eggs absence, *p* = 0.5270). Finally, when body‐shaking was observed, its total number was overall higher in *R. grassei* compared to *R. flavipes* (Figure 3c), whereas it was independent of eggs and descendants presence, as well as of the type of donor (Table 3b).

**Table 3:** Effects of donor, species, eggs and the presence of descendants on (a) the presence/absence and (b) total number of body‐shaking

	(a) Presence/absence		(b) Number of event when expressed	
	LR $\chi_{1}^{2}$	*p*	LR $\chi_{1}^{2}$	*p*
Donor	0.0	0.9390	0.3	0.5943
Species	0.0	0.9350	4.5	**0.0347**
Eggs presence (Ep)	0.3	0.6051	1.3	0.2457
Descendants presence (Dp)	4.5	**0.0348**	0.0	0.9506
Ep:Dp	5.0	**0.0259**	2.4	0.1189
Donor:Ep	6.8	**0.0090**	0.4	0.5395

Note

Significant *p*‐values are in bold.

## 4 DISCUSSION

In this study, we compared the involvement of queens and kings in social interactions during colony foundation in the invasive *R. flavipes* and the native *R. grassei* termites. Our results first reveal that queens invest more in the measured forms of parental care than kings, as they overall performed more trophallaxis, grooming, and antennation (when descendants are present) than their partner. This sex‐specific effect was independent of the species. Secondly, we showed that differences in parental care are species specific*.* In particular, *R. flavipes* exhibited less grooming/antennation toward eggs compared to partners, but more grooming/antennation toward descendants compared to partners. By contrast, *R. grassei* exhibited more grooming/antennation toward partners compared to both eggs and descendants. The two species also differed in their overall expression of stomodeal trophallaxis, which was more likely to occur in *R. flavipes *compared to *R. grassei*. This difference was absent in term of proctodeal trophallaxis. In both species, trophallaxis was preferentially directed toward descendants instead of partners and was overall more likely to involve proctodeal instead of stomodeal contacts. Finally, our results reveal that body‐shaking depends on the species, the sex of the donor, and the developmental stage of the offspring. Body‐shaking was overall more frequent in *R. grassei* compared to *R. flavipes*. Moreover, queens were more likely to perform body‐shaking in the absence compared to presence of eggs, whereas this effect was absent in kings. When eggs were present, body‐shaking was also more likely to occur in the presence compared to absence of descendants, whereas this effect was not found in the absence of eggs.

Somewhat surprisingly, our results reveal that queens and kings exhibit unbalanced investment in the measured forms of parental care during colony foundation in both *R. flavipes *and *R. grassei*. In particular, the involvement of queens into grooming/antennation and trophallaxis was overall higher than the one of kings. This finding both contrasts with the few results reporting an absence of sexual polyethism in incipient colonies of two other termite species, *Zootermopsis angusticollis* and *Z. nevadensis *(Rosengaus & Traniello, 1991; Shellman‐Reeve, 1990), and provides no support for the general prediction of a tight association between lifelong monogamy and balanced investment of each parent into egg/offspring care (Boomsma, 2009). The higher investment of queens into direct interactions in both *R. grassei *and *R. flavipes* suggests that kings are either involved into other tasks and/or overall less active than queens during colony foundation. The success of colony foundation generally involves a broad set of tasks, such as nest construction and/or fights against predators and pathogens (Chouvenc, Efstathion, Elliott, & Su, 2013; Eggleton, 2010), or could be a dynamic response to local environmental changes (Shellman‐Reeve, 1990), for which kings might indeed be more involved. The full task repertoire exhibited by termite queens and kings during colony foundation will be investigated in the future experiments taking into account other life traits like sexual size dimorphisms, physiological traits, or metabolite composition which might be part of the observed unbalanced biparental care.

While both *R. flavipes* and *R. grassei *show unbalanced levels of parental care, we found species‐specific levels of parental investment for grooming/antennation depending on the presence of eggs or descendants. In particular, if we compare the duration of grooming/antennation toward eggs/descendants with the one toward partners (for both species), then *R. grassei* exhibited identical durations in the presence of eggs and descendants, whereas *R. flavipes* exhibited less grooming/antennation in the presence of eggs compared to in the presence of descendants. In social insects, grooming and antennation typically increase the development and survival of offspring (larvae and eggs) by mediating the application of chemical compounds preventing the risks of desiccation or microbial infections (Bulmer, Denier, Velenovsky, & Hamilton, 2012; Fujita, Minamoto, Shimizu, & Abe, 2002; Matsuura et al., 2002), by mechanically removing external pathogens from the cuticles (Rosengaus, Maxmen, Coates, & Traniello, 1998) and by facilitating ecdysis or egg hatching (Whitman & Forschler, 2007). It also allows to directly assess the nestmates needs and also increase social cohesion through exchange of chemical cues (Blomquist & Bagnères, 2010; Hoffmann, Gowin, Hartfelder, & Korb, 2014; Lucas et al., 2018; Lucas, Pho, Fresneau, & Jallon, 2004, 2005 ; Soroker et al., 2003). The reported differences of grooming/antennation between *R. flavipes* and *R. grassei *suggest a species‐specific role of parental care in the success of colony foundation, which might explain the differences in the colony foundation success observed between those two species (Brossette et al., 2017; Leniaud et al., 2011). Further studies should nevertheless be conducted to investigate whether the intrinsic quality of eggs and juveniles differ between *R. flavipes* and *R. grassei*, and whether parental care can mitigate the costs of these intrinsic differences in terms of foundation success.

Our results also reveal that parents exhibited more trophallaxis with their descendants compared to their partners, independent of both the sex of the parent and the species. In subterranean termites such as *R. flavipes* and *R. grassei*, trophallaxis between adults and juveniles typically mediate the transmission of symbionts that are necessary to digest wood (Fujita, Shimizu, & Abe, 2001). These symbionts are present in adults, but generally absent in newborn descendants (Nalepa, Bignell, & Bandi, 2001). Our finding thus suggests that both queens and kings are equally involved into the transmission of symbionts to the descendants in *R. flavipes* and *R. grassei*. Interestingly*, *our results also shed light on the occurrence of trophallaxis between parents. In addition to its potential role in the homogenization of gut microbial community among parents (Nalepa et al., 2001), this occurrence may also mediate the regular exchange of nutrients (particularly for larvae instars which are unable to feed themselves; Nalepa & Jones, 1991), nestmate recognition cues (Kirchner & Minkley, 2003; Soroker et al., 2003), and/or immune defenses between colony members (Bulmer, Bachelet, Raman, Rosengaus, & Sasisekharan, 2009; Chouvenc, Su, & Robert, 2009; Mirabito & Rosengaus, 2016). Our results also suggest that the nature of the compounds exchanged could be driven by the mode of transfer (proctodeal vs. stomodeal) and/or the donor (queens vs. kings). The absolute quantities of the fluids transferred are unknown; thus, the exact investment of each parent is difficult to assess.

Although body‐shaking has been described as a response to disturbance in a large number of termites (Bagnères & Hanus, 2015; Howse, 1965), the modality of its expression remained unclear (Funaro et al., 2018) and was not previously studied in incipient colonies (Rosengaus & Traniello, 1991; Shellman‐Reeve, 1990). Our results reveal that body‐shaking is a relatively frequent behavior exhibited by both parents at colony foundation and that its expression depends on the species, the sex of the parent and the presence/absence of eggs and descendants. In particular, body‐shaking was overall more frequent in *R. grassei* compared to *R. flavipes,* in the presence compared to the absence of descendants, and finally less frequently expressed by queens in the presence compared to absence of eggs. Termites are known to use vibration communication to quickly transmit information thorough the entire colony (Hunt & Richard, 2013). Body‐shaking might be part of this communication system and mediates the rapid spread of a social signal. The importance of egg presence on its expression suggests that the body‐shaking might be used to transmit information on the reproductive state of the incipient colony to the other member of the colony, either independently or in complement with other potential chemical signals. Indeed, the presence of eggs or descendants could represent a proxy of the reproductive state of the incipient colony that could modulate social organization. More investigations are needed to fully explain the observed interactions between the body‐shaking and the presence of eggs or descendants and to explore all factors possibly involved in its expression. Those studies would also allow to explain why this behavior was conserved over several termite species (Bagnères & Hanus, 2015).

Overall, this study sheds light on unbalanced investment into parental care by queens and kings during colony foundation, as well as on species‐specific patterns of social interactions between the invasive *R. flavipes* and the native *R. grassei* termites. These findings emphasize that despite lifelong monogamy, the two parents are not equally involved in the measured forms of parental care and instead suggest that kings are specialized in other tasks and/or overall less active. Second, the presence of species‐specific patterns of social interactions may provide important insights into our understanding of the invasive success of *R. flavipes *(Brossette et al., 2017; Perdereau et al., 2010; Perdereau, Dedeine, Christidès, Dupont, & Bagnères, 2011). More generally, the sex‐specific organization of parental care during termites’ colony foundation emphasizes that males could play a central, yet poorly studied role in the evolution and maintenance of the eusocial organization.

## CONFLICT OF INTEREST

The authors declare that they have no conflict of interest.

## AUTHOR CONTRIBUTIONS

The experiment was designed by CL, LB, and AGB. SD, CL, and LB performed the field work. LB, CL, and SD performed the termite crossings and transferred royal couples to the glass cases. LB followed colony development and analyzed the movies. JM, LB, and CL conducted the statistical analyses. JM, CL, LB, and AGB wrote the manuscript. All the authors read and approved the final version of the manuscript.

## DATA ACCESSIBILITY

Data available from the Dryad Digital Repository: https://doi.org/10.5061/dryad.vs6md76
